# KLF4 promotes milk fat synthesis by regulating the PI3K-AKT-mTOR pathway and targeting FASN activation in bovine mammary epithelial cells

**DOI:** 10.1016/j.isci.2024.109850

**Published:** 2024-04-30

**Authors:** Hong-Yu Wu, Zhong-Hao Ji, Wen-Yin Xie, Hai-Xiang Guo, Yi Zheng, Wei Gao, Bao Yuan

**Affiliations:** 1 Department of Laboratory Animals, College of Animal Sciences, Jilin University, Changchun, Jilin 130062, China; 2 Department of Basic Medicine, Changzhi Medical College, Changzhi 046000, Shanxi, China; 3 Jilin Academy of Agricultural Sciences, Jilin 132101, China

**Keywords:** dairy cattle molecular biology, animal physiology, molecular biology, animal food products

## Abstract

Milk fat is an important indicator for evaluating the quality of cow’s milk. In this study, we used bovine mammary epithelial cells (BMECs) to investigate the role and molecular mechanism of KLF4 in the regulation of milk fat synthesis. The results showed that KLF4 was more highly expressed in mammary tissues of high-fat cows compared with low-fat cows. KLF4 positively regulated the expression of genes related to milk fat synthesis in BMECs, increasing intracellular triglycerides content, and KLF4 promoted milk fat synthesis by activating the PI3K-AKT-mTOR signaling pathway. Furthermore, the results of animal experiments also confirmed that knockdown of KLF4 inhibited milk fat synthesis. In addition, yeast one-hybrid assays and dual-luciferase reporter gene assays confirmed that KLF4 directly targets and binds to the fatty acid synthase (FASN) promoter region to promote FASN transcription. These results demonstrate that KLF4 is a key transcription factor for milk fat synthesis in BMECs.

## Highlights

• KLf4 promotes milk fat synthesis in bovine mammary epithelial cells
• KLF4 regulates the PI3K-AKT-mTOR signaling pathway
• KLF4 directly targets the FASN promoter region

## Graphical abstract

**Figure:**
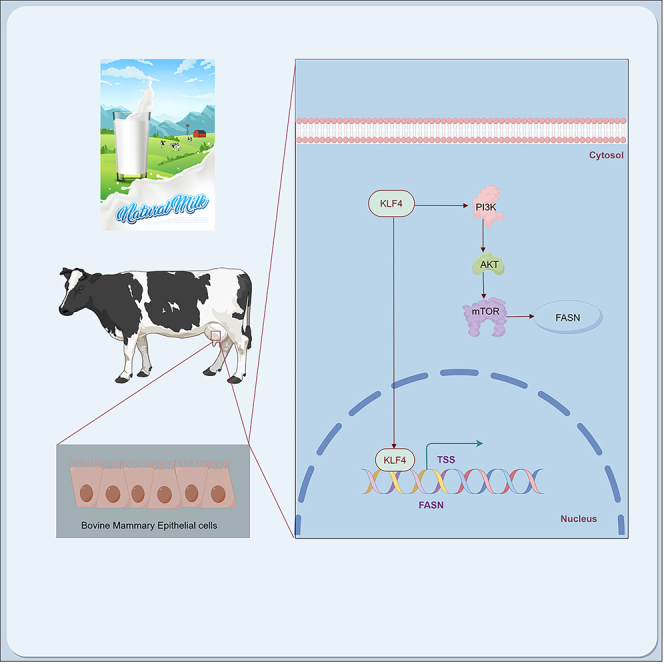

## Introduction

Milk is rich in nutrients, and its consumption has several benefits for the organism, including growth promotion, modulation of immune response, prevention of gastrointestinal infections, and regulation of intestinal microflora.^1^^,^^2^^,^^3^ Milk fat, lactose, and whey protein are the main nutrients in milk, of which milk fat is a natural fat produced by the mammary glands of dairy cows and is an important indicator for evaluating milk quality.^4^ Mammary epithelial cells (MECs) are the functional unit of lactation in dairy cows' mammary glands, and the proliferative capacity of MECs and the ability to synthesize milk fat directly affect mammary gland development and lactation. Therefore, it is very important to investigate the regulatory mechanism of milk fat synthesis in MECs to improve milk quality.

Milk lipid synthesis is a physiological reaction encompassing multiple steps, and the processes of fatty acid uptake and transport, triglyceride synthesis, and lipid droplet formation and secretion are finely regulated by numerous molecules.^5^ Krüppel-like factor 4 (KLF4) can promote lipid synthesis via SREBP1.^6^ In bovine MECs (BMECs), Sterol regulatory element-binding protein 1 (SREBP1) is a key transcription factor regulating cholesterol synthesis and lipid homeostasis,^7^^,^^8^ and it can promote the expression of a variety of cholesterol and lipogenic genes after entering the nucleus.^8^^,^^9^ Activation of the phosphatidylinositol 3-kinase (PI3K)-protein kinase B(AKT)-mammalian target of rapamycin (mTOR) signaling axis promotes adipogenesis via SREBP1.^10^ SREBP1 is a regulator of fatty acid synthase (FASN) gene expression^11^; knockdown of SREBP1 decreases the expression of FASN.^12^ FASN regulates fatty acid synthesis from scratch and is one of the key rate-limiting enzymes of fat synthesis, and its gene expression is positively correlated with milk fat content and milk production in cows.^13^^,^^14^

KLF4 is an evolutionarily conserved zinc-finger structural domain-containing transcription factor that regulates gene transcription by binding to the 5′-CACCC-3′ motif. Available studies have shown that KLF4 is involved in a variety of physiological and pathological processes, such as the regulation of macrophage polarization,^15^ astrocyte activation,^16^ and skin and renal fibrosis.^17^^,^^18^ In early porcine embryos, overexpression of KLF4 promotes chromatin decondensation and accumulation of lipid droplets^19^; in mouse 3T3-L1 precursor adipocytes, KLF4-targeted activation of CCAAT-enhancer-binding protein(C/EBPβ) promotes lipid synthesis^20^; and in human immortalized sebocytes, KLF4-targeted activation of C/EBPβ and SREBP1 promotes lipid synthesis,^6^ whereas, in goat intramuscular preadipocytes, KLF4 was shown to inhibit lipogenic differentiation by targeting C/EBPβ.^21^ In BMECs, the relationship between KLF4 and milk fat synthesis is unclear.

Therefore, the aim of this study was to analyze the regulatory role and potential mechanisms of KLF4 in milk fat synthesis. The findings will expand our understanding of the regulatory network of milk fat synthesis in BMECs.

## Results

### KLF4 is differentially expressed in mammary tissues of high- and low-fat cows

**Figure 1:**
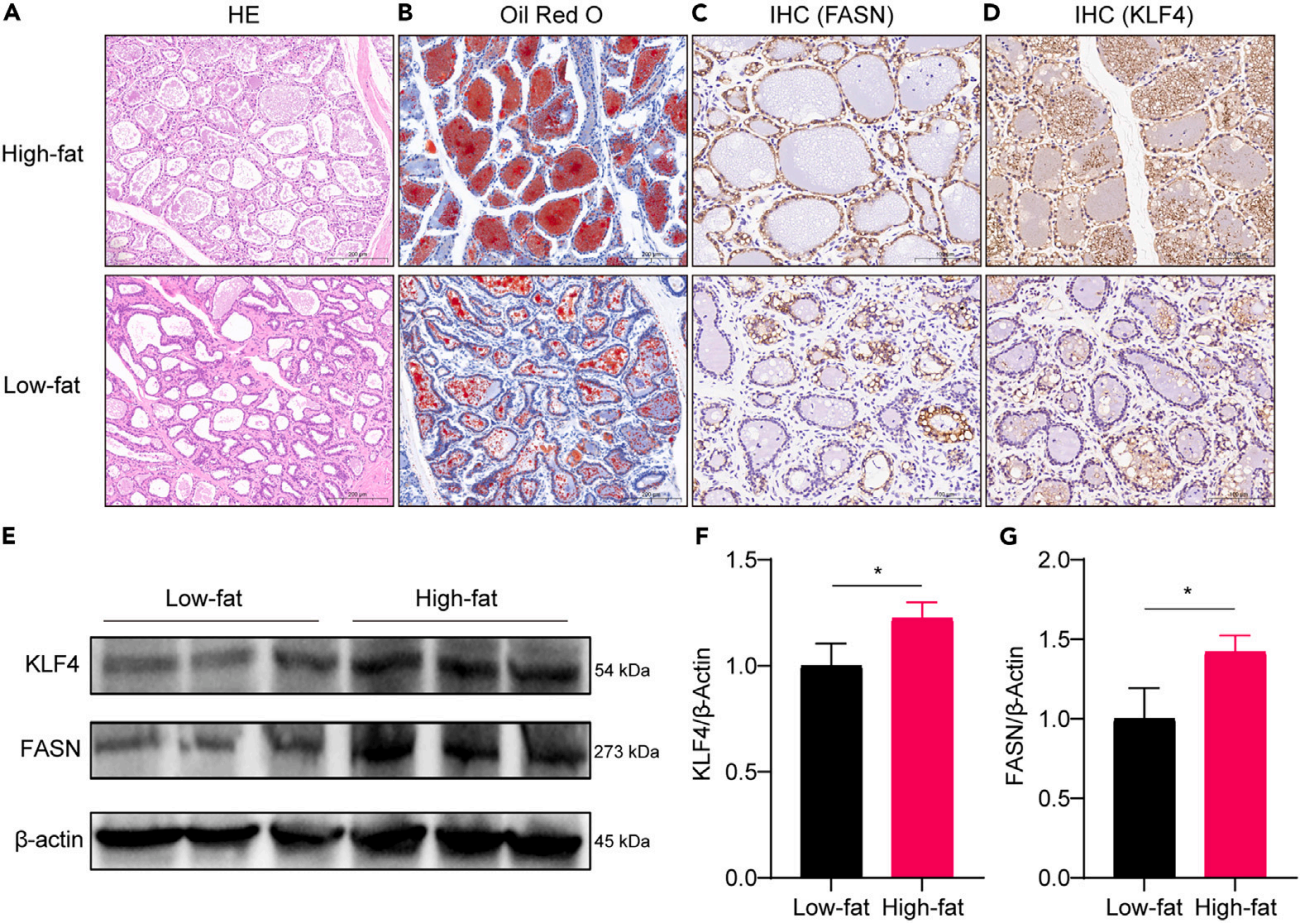
KLF4 is highly expressed in mammary tissues of high-milk-fat cows (A) HE staining; (B) oil red O staining; (C) immunohistochemistry (IHC) detection of FASN protein expression in mammary tissues; (D) IHC detection of KLF4 protein expression in mammary tissues; (E) western blot (WB) detection of FASN and KLF4 protein expression in mammary tissues; (F) KLF4 protein quantitative analysis results; (G) quantitative analysis results of FASN protein. (*n* = 3) ∗*p* < 0.05.

The morphology of the mammary alveoli and fat deposition in mammary tissues were analyzed by H&E staining and oil red O staining, which showed that the mammary alveoli in high-fat cows were fuller and the amount of fat deposition was significantly higher than that in low-fat cows (Figures 1A and 1B). Immunohistochemistry (IHC) and western blotting were used to examine the expression of FASN and KLF4 in mammary tissues of both high-fat and low-fat dairy cows. The results showed that the protein expression levels of both FASN and KLF4 were significantly higher in high-milk-fat cows than in low-milk-fat cows (Figures 1C–1G). The above results suggest that KLF4 expression may be related to milk fat synthesis.

### Overexpression of KLF4 in BMECs promotes milk fat synthesis

**Figure 2:**
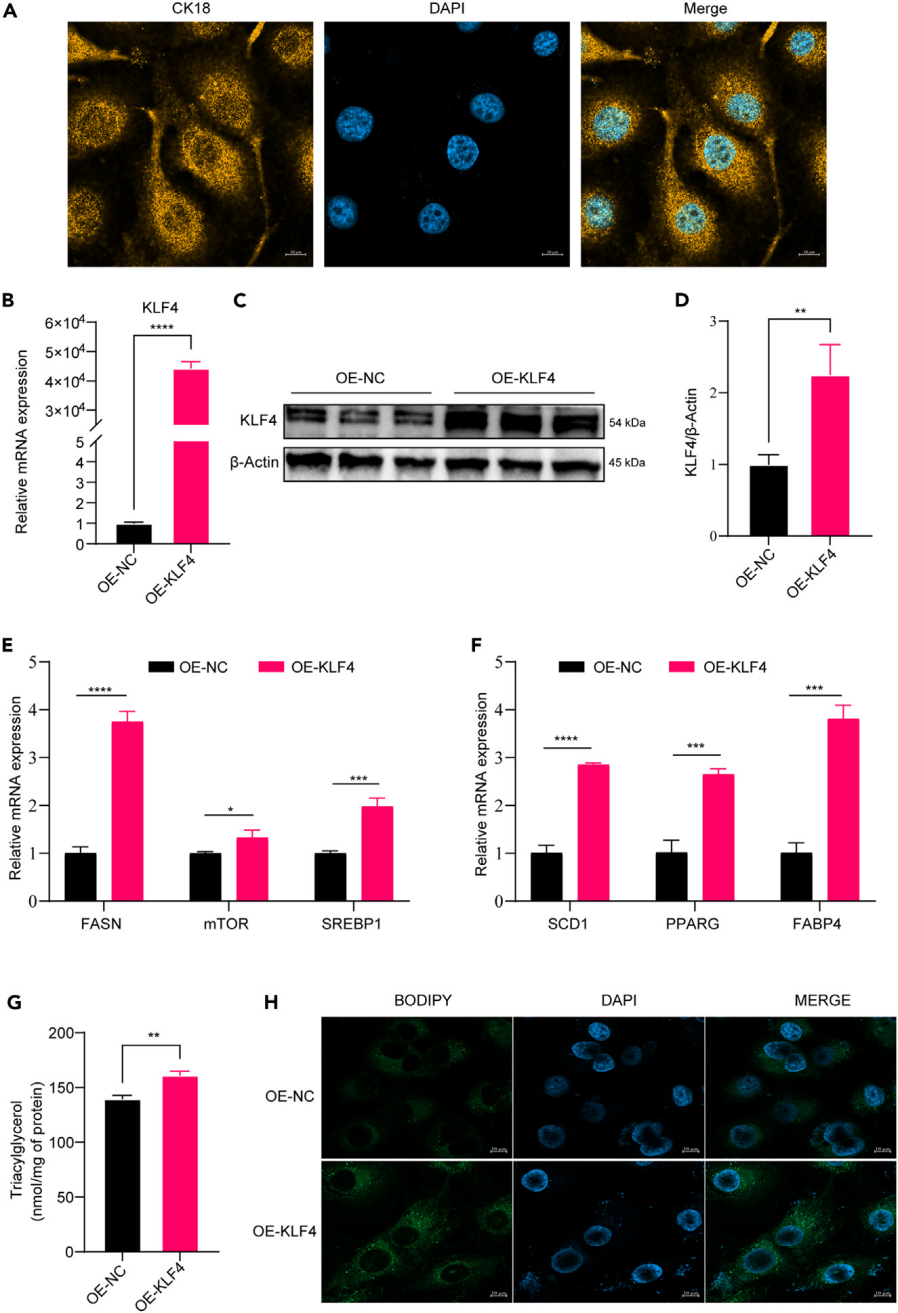
Overexpression of KLF4 in bovine mammary epithelial cells (BMECs) promotes milk fat synthesis (A) Immunofluorescence detection of CK18 for the identification of BMECs; (B–D) RT-qPCR and WB to verify the overexpression efficiency of KLF4; (E and F) RT-qPCR to detect the mRNA expression of genes related to milk lipid synthesis; (G) the content of intracellular triglycerides; (H) BODIPY staining to observe the lipid droplets in the package; green indicates the location of lipid droplets, and blue indicates the location of the cell nucleus. (*n* = 3) ∗*p* < 0.05, ∗∗*p* < 0.01, ∗∗∗*p* < 0.001, ∗∗∗∗*p* < 0.0001.

Immunofluorescence analysis was performed to observe the expression of CK18 in BMECs to exclude contamination by fibroblasts (Figure 2A). Overexpression of KLF4 was manipulated by transfecting plasmids into BMECs to analyze the effect of KLF4 on milk lipid synthesis. The overexpression efficiency was verified by quantitative reverse-transcription PCR (RT-qPCR) and western blot detection of KLF4 expression levels (Figures 2B–2D). The results of functional experiments showed that the mRNA expression levels of the genes related to milk lipid synthesis, FASN, mTOR, SERBP1, Stearoyl-CoA desaturase1 (SCD1), Peroxisome proliferator-activated receptor gamma (PPARG), and Fatty acid binding protein-4 (FABP4) were significantly increased after overexpression of KLF4 (*p* < 0.05) (Figures 2E and 2F), and the triglycerides content in the cells was significantly increased (*p* < 0.01) (Figure 2G). BODIPY staining results showed that overexpression of KLF4 promoted lipid droplet formation (Figure 2H). The aforementioned results indicated that KLF4 overexpression in BMECs promoted milk fat synthesis.

### Knockdown of KLF4 in BMECs inhibits milk fat synthesis

**Figure 3:**
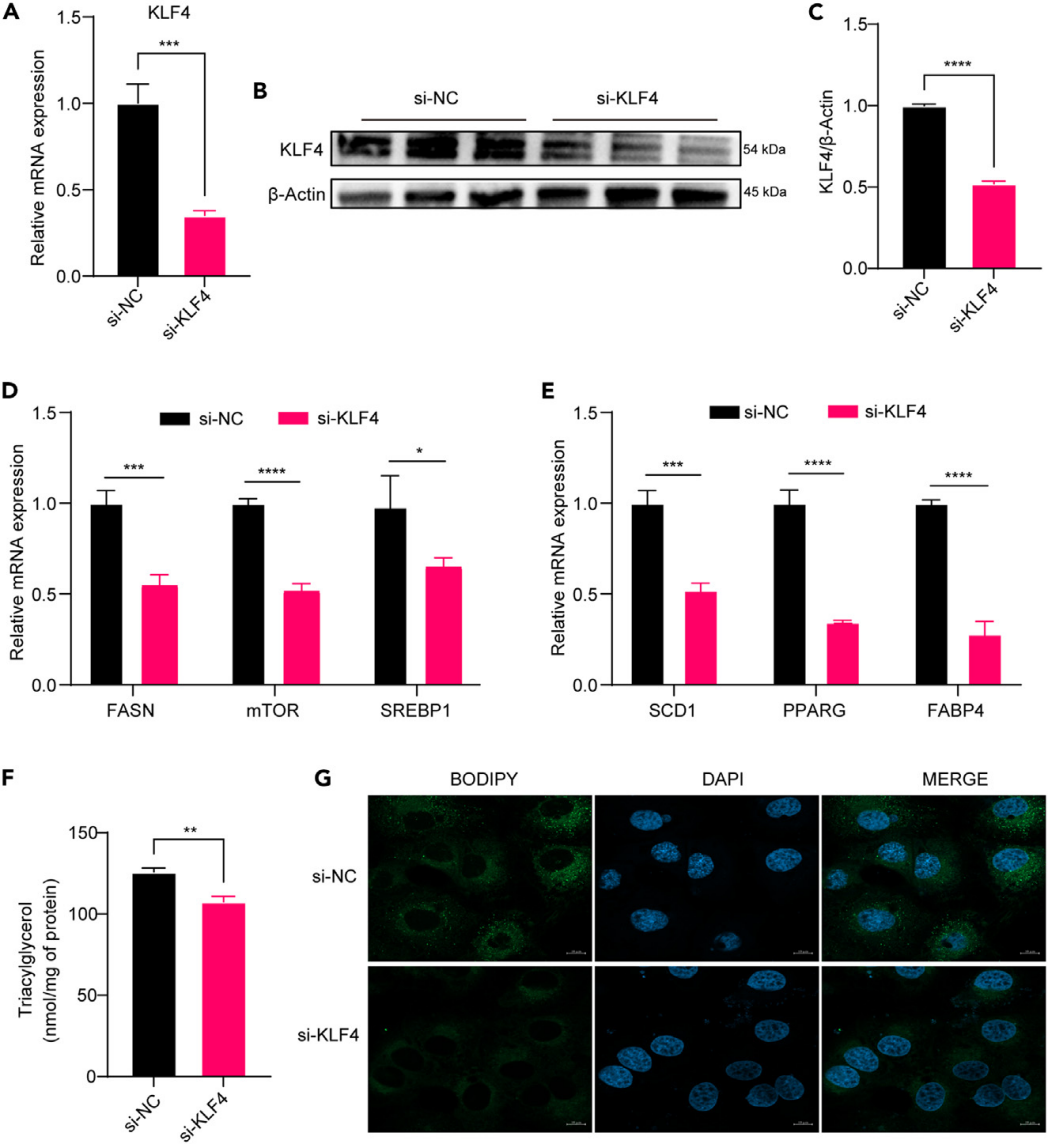
Knockdown of KLF4 in bovine mammary epithelial cells (BMECs) to inhibit milk fat synthesis (A–C) RT-qPCR and WB to verify the knockdown efficiency of KLF4; (D and E) RT-qPCR to detect the mRNA expression of the genes related to milk fat synthesis; (F) the content of intracellular triglycerides; (G) BODIPY staining to observe the intracellular lipid droplets, with green indicating the location of the droplets and blue indicating the location of the nucleus. Blue indicates the position of the cell nucleus. (*n* = 3) ∗*p* < 0.05, ∗∗*p* < 0.01, ∗∗∗*p* < 0.001, ∗∗∗∗*p* < 0.0001.

A small interfering RNA (siRNA) sequence targeting KLF4 was designed to inhibit KLF4 expression in MECs transfected with si-KLF4 to further clarify the regulatory role of KLF4 in milk lipid synthesis (Figures 3A–3C). The efficiency of the knockdown was verified by RT-qPCR and western blot analysis of the KLF4 expression level. After KLF4 knockdown, the mRNA expression levels of milk fat synthesis-related genes FASN, mTOR, SERBP1, SCD1, PPARG, and FABP4 were significantly reduced (*p* < 0.05) (Figures 3D and 3E), and the triglycerides content of the cells was significantly reduced (*p* < 0.01) (Figure 3F). BODIPY staining results showed that knockdown of KLF4 inhibited lipid droplet formation (Figure 3G). Taken together, the aforementioned experimental results demonstrated that KLF4 positively regulated milk lipid synthesis in MECs of dairy cows.

### KLF4 promotes milk fat synthesis through the PI3K-AKT-mTOR-FASN signaling axis

**Figure 4:**
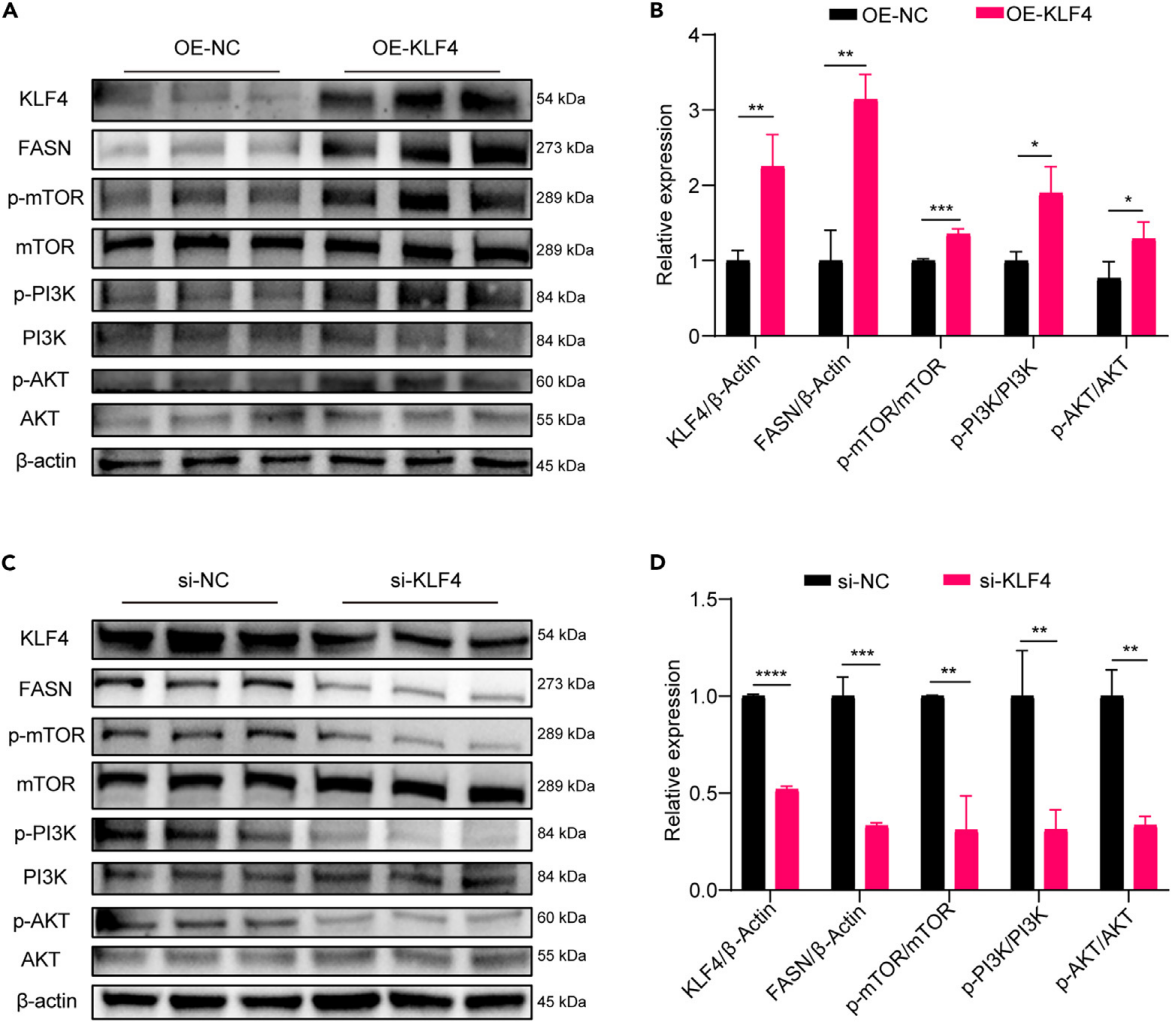
KLF4 positively regulates the PI3K-AKT-mTOR-FASN signaling axis (A and B) Western blot (WB) assay after KLF4 overexpression to analyze the changes in KLF4 and total FASN protein expression and changes in the levels of PI3K, AKT, and mTOR protein phosphorylation; (C and D) WB assay after knockdown of KLF4 to analyze the changes in the expression of KLF4 and total FASN protein and the changes in the levels of phosphorylation of PI3K, AKT, and mTOR proteins. (*n* = 3) ∗*p* < 0.05, ∗∗*p* < 0.01, ∗∗∗*p* < 0.001, ∗∗∗∗*p* < 0.0001.

The PI3K-AKT-mTOR signaling pathway has been widely reported to be involved in the regulation of cellular lipid metabolism.^22^^,^^23^ To explore the mechanism by which KLF4 regulates milk lipid synthesis, western blot experiments were used to analyze the effects of KLF4 overexpression or knockdown on the phosphorylation of PI3K, AKT, and mTOR proteins. The results showed that, after overexpression of KLF4, the expression of KLF4 and FASN proteins was significantly increased (*p* < 0.01), and the levels of mTOR, PI3K, and AKT protein phosphorylation also significantly increased (*p* < 0.05) (Figures 4A and 4B); consistent with these results, knockdown of KLF4 resulted in a significant decrease in the expression of KLF4 and FASN (*p* < 0.001) proteins and in the levels of mTOR, PI3K, and AKT protein phosphorylation (*p* < 0.01) (Figures 4C and 4D).

**Figure 5:**
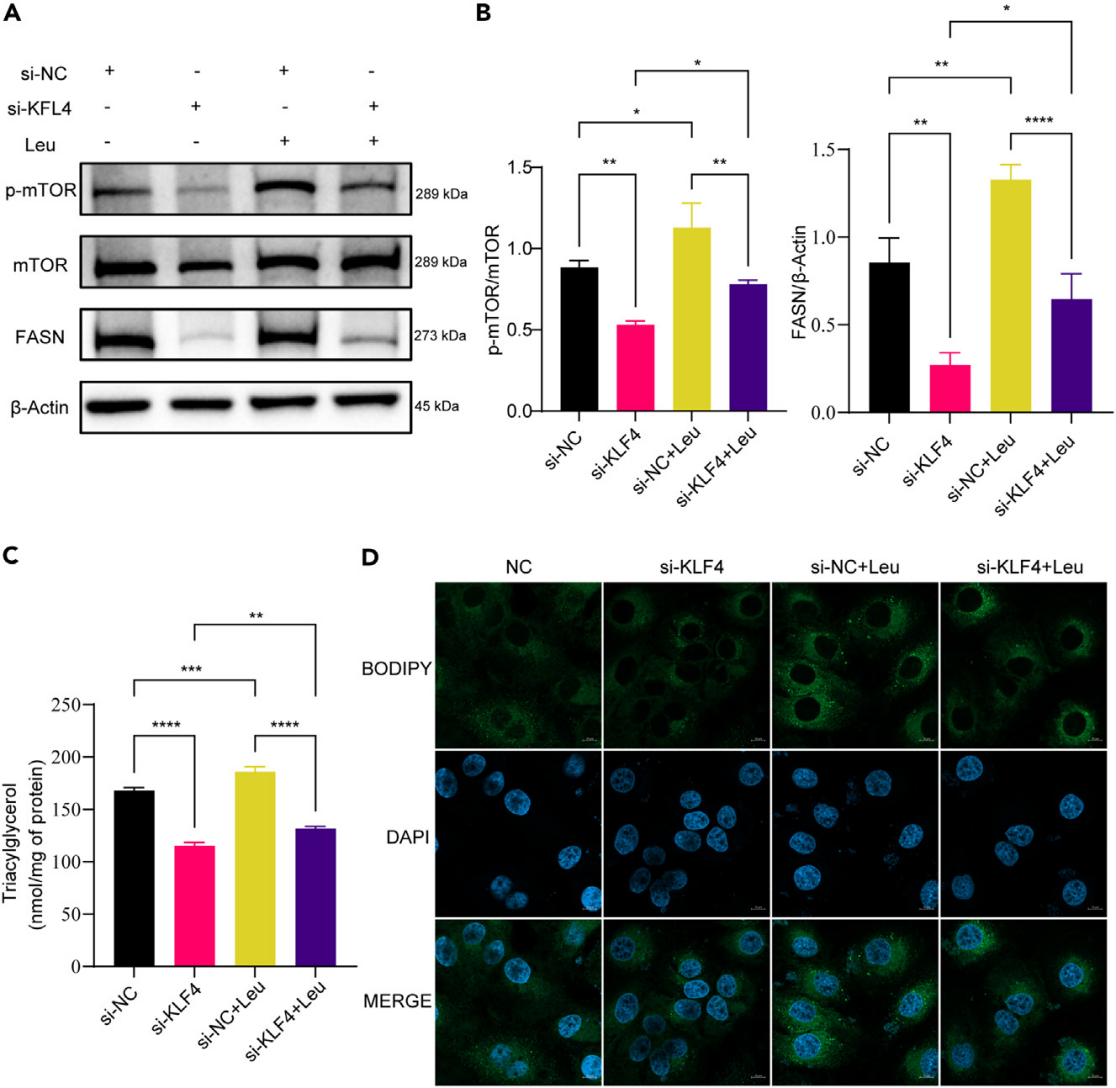
The promotion of milk fat synthesis by KLF4 is dependent on mTOR activation (A and B) Western blot (WB) detection of the protein expression of FASN and mTOR after different treatments; (C) kit detection of triglycerides content; (D) BODIPY staining to observe intracellular lipid droplets. (*n* = 3) ∗*p* < 0.05, ∗∗*p* < 0.01, ∗∗∗*p* < 0.001, ∗∗∗∗*p* < 0.0001.

To further clarify the necessity of mTOR activation for KLF4 to regulate milk lipid synthesis, Leucine (Leu) was added to activate mTOR while knocking down KLF4 to analyze whether the regulatory effect of KLF4 on milk lipid synthesis was altered. Western blot results showed that the level of phosphorylated mTOR protein was elevated after Leu treatment and that the inhibitory effect of si-KLF4 on FASN protein and mTOR protein phosphorylation was partially reversed by Leu (*p* < 0.05) (Figures 5A and 5B). Triglycerides assay results and BODIPY staining results showed that Leu was able to partially reverse the inhibitory effect of si-KLF4 on lipid synthesis and lipid droplet formation (Figures 5C and 5D). The aforementioned results suggest that KLF4 promotes milk lipid synthesis through the PI3K-AKT-mTOR-FASN signaling axis.

### Targeted binding of KLF4 to the promoter region of FASN

**Figure 6:**
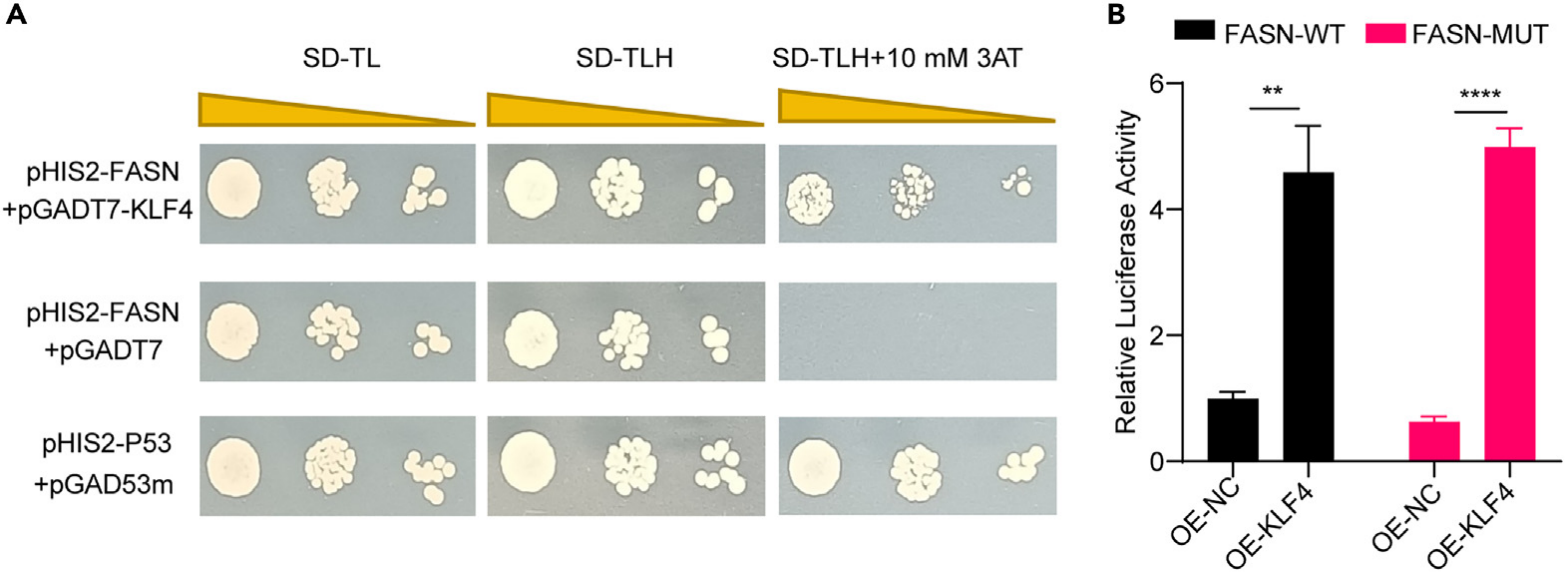
KLF4 targets binding to the promoter region of FASN to promote its transcription (A) Yeast one-hybrid point-to-point validation of the interactions between KLF4 proteins and the promoter region of FASN; (B) dual-luciferase reporter gene assay to analyze the interactions between the KLF4 proteins and the FASN promoter. ∗∗*p* < 0.01, ∗∗∗∗*p* < 0.0001.

KLF4 is a transcription factor with the classical binding motif CACCC, and database queries showed multiple KLF4 binding sites within 2,000 bp upstream of the transcription start site of FASN. Therefore, we speculate that KLF4 may directly transcriptionally activate FASN expression. Yeast one-hybrid assays and dual-luciferase reporter gene assays were used to analyze whether the KLF4 protein binds to the FASN promoter region. The results of the yeast one-hybrid assay showed that positive controls, as well as yeast transfected with the pHIS2- FASN bait vector and the pGADT7-KLF4 prey vector, were able to survive in the defective medium supplemented with amino-1,2,4-triazole (3AT), a competitive inhibitor of the yeast HIS3 protein used to inhibit self-activating effects in yeast, whereas negative controls transfected with an empty vector were not able to survive, suggesting binding occurs between the KLF4 protein and the FASN promoter region (Figure 6A). The results of the dual-luciferase reporter gene assay showed that the luciferase activities of both FASN-WT (wild type) and FASN-MUT (mutation) were significantly elevated after KLF4 overexpression (*p* < 0.01) (Figure 6B). These results suggest that KLF4 promotes the transcription of FASN by directly binding to its promoter region. Moreover, the 208–601 region upstream of the FASN transcription start site is not a critical region for KLF4 binding to FASN.

### Knockdown of KLF4 at the animal level inhibits milk fat synthesis

**Figure 7:**
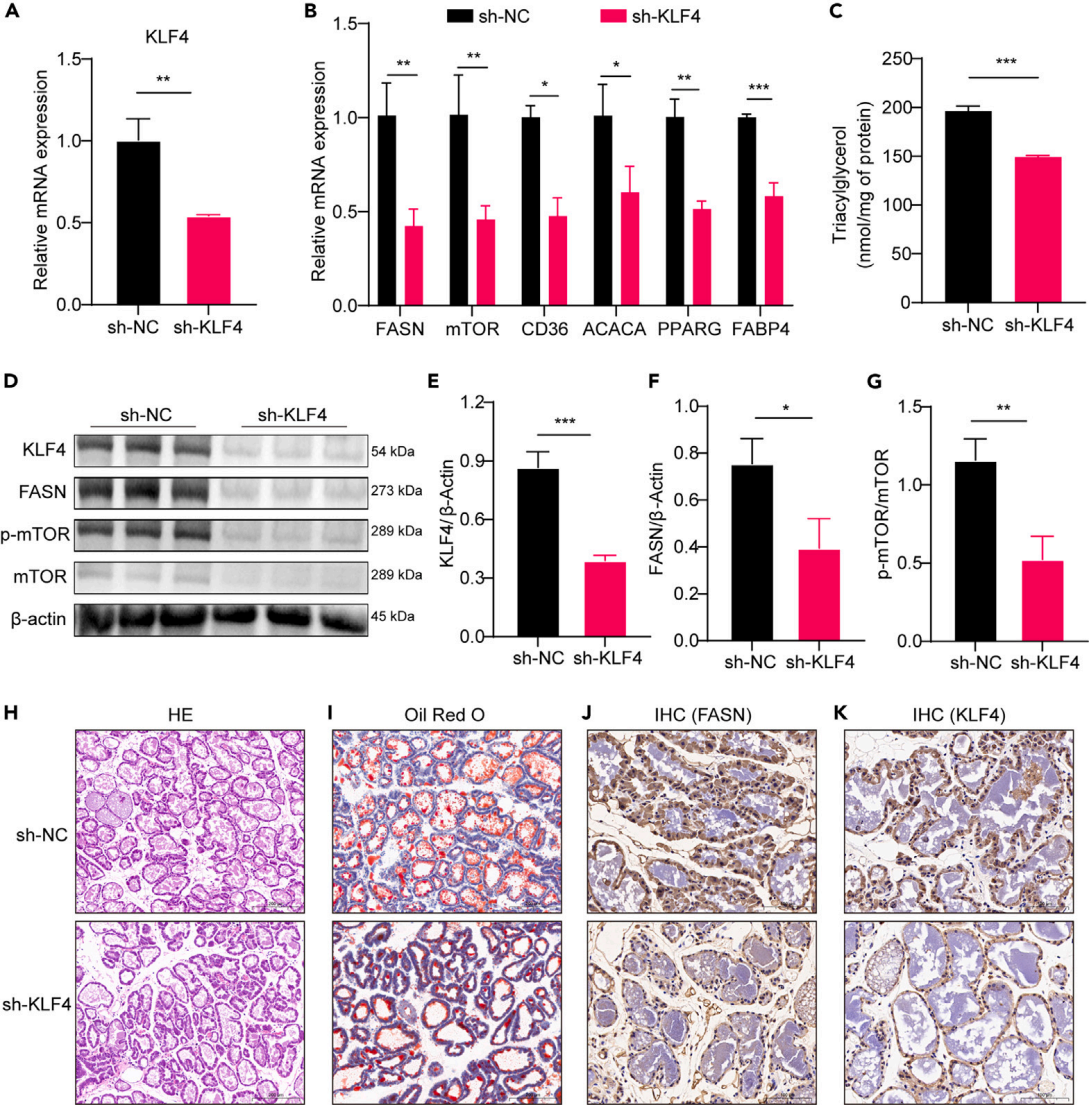
Knockdown of KLF4 to inhibit milk fat synthesis in an *in vivo* mouse experiment (A) RT-qPCR to detect the mRNA expression of KLF4; (B) RT-qPCR to detect the mRNA expression of genes related to milk fat synthesis; (C) tissue triglycerides content; (D–G) western blot (WB) to detect the expression of FASN and KLF4 proteins in mammary gland tissues and the mTOR protein phosphorylation level; (H) H&E staining; (I) oil red O staining; (J) immunohistochemistry (IHC) detection of FASN protein expression in breast tissue; (K) IHC detection of KLF4 protein expression in breast tissue. (n = 3–5) ∗*p* < 0.05, ∗∗*p* < 0.01, ∗∗∗*p* < 0.001.

The regulatory role of KLF4 in milk fat synthesis was analyzed at the whole-animal level. Subcutaneous injection of a lentiviral vector containing shRNA-KLF4 into the fourth mammary gland pair of the mouse inhibited the expression of KLF4, and the knockdown efficiency was verified at the mRNA and protein levels (Figures 7A, 7D, 7E, and 7K). RT-qPCR results showed that the mRNA expression of genes related to milk lipid synthesis was significantly reduced after KLF4 knockdown (*p* < 0.05) (Figure 7B). The triglycerides content in the mammary gland was significantly reduced in the sh-KLF4 group (*p* < 0.001) (Figure 7C). Western blot results showed that the protein expression of FASN was significantly reduced after knockdown of KLF4 (*p* < 0.05) (Figures 7D and F), and the level of mTOR phosphorylation was significantly reduced (*p* < 0.01) (Figures 7D and 7G). The oil red O staining and IHC results were consistent with the finding that the lipid content and FASN protein expression in the mammary gland were lower in the sh-KLF4 group than in the shRNA-NC group. The aforementioned results showed that KLF4 knockdown in the mouse mammary gland inhibited lipid synthesis.

### Effects of KLF4 knockdown on mammary gland metabolism analyzed by LC-MS

**Figure 8:**
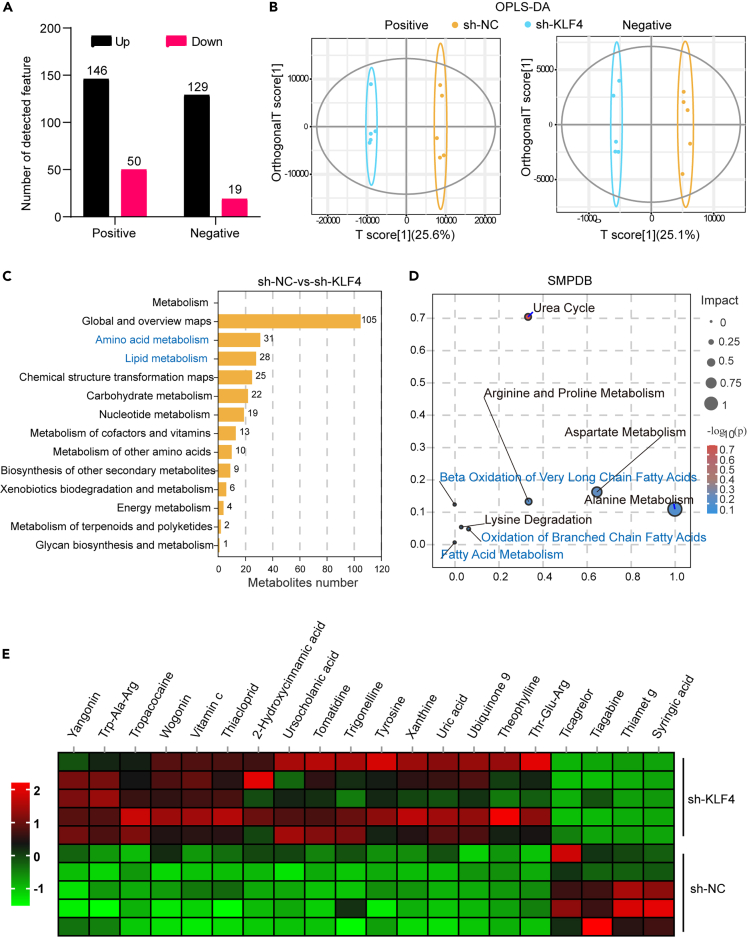
LC-MS analysis of the effect of KLF4 knockdown on mammary gland metabolism (A) Number of differentially abundant metabolites in positive and negative ion modes, with the criteria for determining differences as fold change ≥1.5 or ≤1/1.5, *p* value < 0.05, and VIP value ≥ 1; (B) OPLS-DA downscaling analysis; (C) annotation of KEGG of differentially abundant metabolites; (D) bubble plots of Kyoto Encyclopedia of Genes and Genomes enrichment analysis; (E) heatmap of the *p* value ranked top 20 metabolites heatmap. (*n* = 5).

The effect of KLF4 knockdown on mammary gland metabolism was further assessed by liquid chromatography-mass spectrometry (LC‒MS). With the criteria for determining differences set as fold change ≥1.5 or ≤1/1.5, *p* value < 0.05, and variable important for the projection (VIP) value ≥ 1, 146 metabolites were upregulated and 50 metabolites were downregulated in the sh-KLF4 group compared with the sh-NC group in the positive ion mode, and 129 metabolites were upregulated and 19 metabolites were downregulated in the sh-KLF4 group compared with the sh-NC group in the negative ion mode (Figure 8A). The orthogonal partial least squares discriminant analysis (OPLS-DA) results showed that the mammary glands of the two groups of mice had different metabolic profiles (Figure 8B). The results of functional enrichment analysis suggested that the differentially abundant metabolites were mainly related to amino acid metabolism and lipid metabolism (Figure 8C). Specifically, lipid metabolism-related pathways, such as beta oxidation of very long-chain fatty acids, oxidation of branched chain fatty acids, fatty acid metabolism, arginine and proline metabolism, aspartate metabolism, alanine metabolism, lysine degradation, and other amino acid metabolism pathways, differed between the two groups (Figure 8D). Figure 8E shows the top 20 differentially abundant metabolites in the heatmap format sorted by *p* value.

## Discussion

KLF4 is a member of the Krüppel-like factor family, which regulates a variety of processes, including cell growth, proliferation, and differentiation.^24^^,^^25^^,^^26^ In this study, we found that KLF4 was differentially expressed in mammary samples from high- and low-milk fat cows, suggesting that KLF4 may be involved in the regulation of milk fat synthesis. The Krüppel-like family of transcription factors contains multiple members that play important roles in adipocyte differentiation and lipid metabolism. KLF2 promotes hepatic steatosis in mice by upregulating CD36.^27^ In BMECs, KLF6 promotes milk fat synthesis.^28^ KLF7 and KLF9 have diametrically opposed functions in goat precursor adipocyte differentiation and lipid deposition, where KLF7 promotes precursor adipocyte differentiation to mature cells^29^ and KLF9 inhibits lipid deposition.^30^ In addition, KLF14 and KLF15 have also been reported to be involved in the regulation of lipid metabolism in the heart and skeletal muscle.^31^^,^^32^^,^^33^

To further clarify the role of KLF4 in milk lipid synthesis, we increased and decreased KLF4 expression in BMECs and detected the lipid synthesis-related gene expression level, triglycerides content, and lipid droplet formation to assess the effects on milk lipid synthesis. The results showed that KLF4 promoted milk lipid synthesis in BMECs. KLF4 can regulate cell proliferation by activating the PI3K-AKT-mTOR signaling pathway.^34^^,^^35^ The role of the PI3K-AKT-mTOR signaling pathway in lipid synthesis has been widely reported, which involves the promotion of milk fat synthesis by activating targets such as SREBP1 and SREBP1c.^36^^,^^37^ Therefore, we speculated that KLF4 might promote milk fat synthesis in BMECs by activating the PI3K-AKT-mTOR signaling pathway. The experimental results corroborated our postulation that the phosphorylation levels of PI3K, Akt, and mTOR were elevated after overexpression of KLF4, and the corresponding knockdown of KLF4 decreased the phosphorylation levels of PI3K, Akt, and mTOR. When overexpressing and knocking down KLF4, KLF4 had two western blot bands. Firstly, KLF4 antibody is a knockdown (KD)/knockout (KO)-validated antibody from Proteintech; it has been validated in 113 papers. The problem of two KLF4 bands due to antibody specificity can be excluded. Secondly, since the two KLF4 bands are very close to each other, we speculate that there are post-translational modifications of the KLF4 protein, such as phosphorylation and acetylation. It also offers prospects for follow-up research. Furthermore, by cotreating with the mTOR pathway activator Leu and si-KLF4, we demonstrated that the regulatory effect of KLF4 on milk fat synthesis is partially dependent on mTOR pathway activation.

Considering the properties of KLF4 as a transcription factor, we used yeast one-hybrid and dual-luciferase reporter gene assays and demonstrated that KLF4 can bind to the promoter region of FASN to activate its transcription. It has been shown that acetic acid and β-hydroxybutyric acid induce FASN expression in a cell death-inducing DFFA-like effector C(CIDEC)-C/EBPβ-dependent manner in MECs of dairy cows and that −110 to −50 upstream of the FASN transcriptional start site is a critical region for C/EBPβ binding.^38^ Liver X receptor α (LXRα) was also shown to promote milk fat synthesis by targeting the activation of FASN transcription, and −793 to +232 upstream of the FASN transcription start site was a critical region for LXRα binding.^39^ Based on the database prediction results, we constructed a mutant plasmid in the promoter region of FASN, and the results showed that the deletion of the −208 to −601 region upstream of the transcription start site did not affect KLF4 and FASN binding. It is speculated that this may be caused by the different preferences of the KLF4 protein in recognizing motifs among different species. It is still necessary to redesign the truncated vector to analyze the binding region of the KLF4 protein.

Furthermore, we inhibited KLF4 expression by subcutaneous injection of lentivirus and demonstrated at the animal level that knockdown of KLF4 inhibited milk fat synthesis. In addition, we analyzed the effects of KLF4 knockdown on mammary metabolic pathways by untargeted metabolomics. The effects of KLF4 knockdown on amino acid metabolism and lipid metabolism were revealed. Lysine, leucine, taurine, and methionine can promote milk lipid synthesis through different signaling pathways.^40^^,^^41^^,^^42^^,^^43^ We hypothesized that there is feedback regulation between KLF4 and amino acid metabolism, so is it possible that amino acid supplementation could affect milk fat synthesis by promoting KLF4 expression. We will test this hypothesis in subsequent experiments.

In summary, the present study demonstrated that, in BMECs, KLF4 promotes milk fat synthesis by regulating the PI3K-AKT-mTOR pathway and targeting FASN activation. This study clarifies the function of KLF4 in milk fat synthesis, and the results will expand our understanding of the regulatory network of milk fat synthesis in dairy cows.

### Limitations of the study

Admittedly, there are some shortcomings in this study. Although this study confirms that the transcription factor KLF4 can directly target the FASN promoter region, the binding site is still unknown. In addition, body weights and feed intake were not recorded in the construction of the mouse model, which did not allow for a more complete understanding of the model.

## STAR★Methods

### Key resources table

**Table:** 

REAGENT or RESOURCE	SOURCE	IDENTIFIER
**Antibodies**		

anti-FASN antibody	CST	CAT# 3180; RRID: AB_2100796
anti-β-actin antibody	CST	CAT# 4970; RRID: AB_2223172
anti-rabbit IgG antibody	CST	CAT# 7074; RRID: AB_2099233
anti-CK18 antibody	Abcam	CAT# ab137860; RRID: AB_11155892
anti-PI3K antibody	Affinity	CAT#AF6241; RRID: AB_2835340
anti-p-PI3K p85 alpha (Tyr607) antibody	Affinity	CAT# AF3241; RRID: AB_2834667
anti-pan-AKT1/2/3 antibody	Affinity	CAT# AF6261; RRID: AB_2835121
anti-p-AKT1/2/3(Ser473) antibody	Affinity	CAT# AF0016; RRID: AB_2810275
anti-mTOR antibody	Affinity	CAT# AF6308; RRID: AB_2835169
anti-p-mTOR (Ser2448) antibody	Affinity	CAT# AF3308; RRID: AB_2834727
anti-KLF4 antibody	Proteintech	CAT# 11880-1-AP; RRID: AB_10640807

**Chemicals, peptides, and recombinant proteins**		

DMEM/F12	VivaCell	CAT# C3132
Insulin	Sigma-Aldrich	CAT# I5500
Hydrocortisone	Sigma-Aldrich	CAT# 3867
Lipofectamine™ 2000	Invitrogen	CAT# 11668500
L- Leucine anti-quenching sealer containing DAPI	MedChem Express Beyotime	CAT# HY-N0486 CAT# P0131
Trizol	Invitrogen	CAT# 15596

**Critical commercial assays**		

triglycerides content	Applygen	CAT# E1013
BODIPY 493/503	Thermo Fisher Scientific	CAT# D2191
MonScript™ RTIII All-in-One Mix with dsDNase	Monad	CAT# MR05101M
ChemoHS qPCR Mix	Monad	CAT# MQ00401
Total RNA Extraction Kit	Sevenbio	CAT# SM130

**Experimental models: Organisms/strains**		

Mice:ICR	Liaoning Changsheng Biotechnology Co	N/A

**Other**		

shKLF4:CCAAGAGTTCTCATCTCAA	JintosBiotech	N/A
siRNA-KLf4:CTACACGAAGAGTTCTCAT	Guangzhou Ruibo Biotechnology Co.	N/A

**Recombinant DNA**		

pcDNA3.1-KLF4	Shanghai Sangong Bioengineering Co.	GENE202301310040

**Software and Algorithms**		

GraphPad Prism	GraphPad Software	http://www.graphpad.com
ImageJ	NIH	https://imagej.nih.gov/ij/index.html

**Deposited data**		

Untargeted metabolomic	CNCB	OMIX006289

### Resource availability

#### Lead contact

Further information and requests for resources and reagents should be directed to and will be fulfilled by the Lead Contact,Bao Yuan (yuan_bao@jlu.edu.cn).

#### Materials availability

This study did not generate new unique reagents.

#### Data and code availability

• All untargeted metabolomic data in this study have been deposited to the China National Center for Bioinformation (https://www.cncb.ac.cn/)and accessed via OMIX series login number OMIX006289.
• This paper does not report original code.
• Data reported in this paper will be shared by the lead contact upon request.
• Any additional information required to reanalyze the data reported in this paper is available from the lead contact upon request.

### Experimental model and study participant details

The Holstein cow mammary tissue samples were gifted by Associate Professor Yang Li from the College of Animal Science and Technology, Northeast Agricultural University, and stored in 4% paraformaldehyde for preparation of tissue sections and in at -80°C for protein and RNA analyses.Under the license of the Animal Care and Use Committee of Northeast Agricultural University (NEAUEC20220257).The Holstein cows involved in the experiment were 3-litre females.Healthy Holstein cows participating in the experiment were fed daily concentrated silage grass and ensured adequate water intake. Based on fat content, mammary tissues derived from 10 Holstein cows were divided into a high-milk-fat group (milk-fat content >3.5%) and a low-milk-fat group (milk-fat content <3.5%), with 5 cows in each group (Table S1).

The ICR mice used in the participating experiments were female and male ICR mice, eight weeks old.

#### Animals

Fifteen SPF-grade ICR mice were purchased from Liaoning Changsheng Biotechnology Co. and housed in the barrier facility of Jilin University Laboratory Animal Center. The animal experiment protocol was reviewed and approved by the Animal Ethics and Welfare Committee of Jilin University (SY202305004).

### Method details

#### Cell culture

MECs were isolated from the mammary tissue of Holstein cows.^44^ Tissue blocks were washed several times using D-Hank's balanced salt solution containing dual antibodies as well as 75% alcohol. The tissue blocks were cut into small pieces using scissors, inoculated in cell culture flasks at an appropriate density and placed in a 37°C incubator with 5% CO_2_. The culture medium was changed every 48 h. The composition of the culture medium was DMEM/F12(C3132,VivaCell) containing 20% fetal bovine serum, 1% double antibiotic, bovine insulin (5 μg/mL,I5500,Sigma-Aldrich), and hydrocortisone (1 μg/mL,3867,Sigma-Aldrich) MECs were digested in stages using 0.25% trypsin to purify MECs according to the different sensitivities of MECs and fibroblasts to trypsin. Characterization of BMECs was achieved using CK18.

#### Animal experiments

After one week of acclimatization, the cages were combined at a ratio of 2 females: 1 male, the female mice were at embryonic stage 0.5 days at the time of the appearance of vaginal plugs, and the female rats were randomly divided into the sh-NC group and the sh-KLF4 group after the separation of males and females into 5 groups each. Lentivirus loaded with sh-KLF4 was injected subcutaneously into the fourth pair of mammary glands on embryonic day 5.5. The sh-NC group was injected with lentivirus encapsulated in an empty vector at a lentiviral titer of 1.5 × 10^8^ plaque forming units per mL, and each mammary gland was injected with 50 μL of the lentiviral. The design, synthesis and lentiviral packaging of sh-KLF4 were performed by Jintos Biotech (Wuhan, China), (sh-KLF4: CCAAGAGTTCTCATCTCAA). On day 5 of lactation, female mice were euthanized by excess carbon dioxide treatment, and the fourth pair of mammary glands was collected. Samples for tissue section preparation were stored in 4% paraformaldehyde, and samples for protein and RNA assays were stored at −80°C for backup.

#### Cell transfection and treatment

The KLF4 overexpression plasmid was constructed by Shanghai Sangong Bioengineering Co., Ltd (Shanghai, China), and the vector used was pcDNA3.1. siRNA for KLF4 was designed and synthesized by Guangzhou Ruibo Biotechnology Co.(si-KLF4: CTACACGAAGAGTTCTCAT). Overexpression plasmids and siRNAs were introduced into cells by transfection using Lipofectamine™ 2000 (Thermo Fisher Scientific, Waltham, MA, USA), and the control group was transfected with the empty plasmid or si-NC sequence. When the cell confluence reached 60%-70%, KLF4 was overexpressed or knocked down. Cell samples were collected for subsequent analysis after 24 h. The mTOR activator L-leucine (Leu) was purchased from MedChem Express (HY-N0486). After siRNA transfection for 6 h, 0.6 mM L-leucine was added to continue treating cells for 24 h, and cell samples were collected for subsequent assays.

#### RT‒qPCR and WB

Total cellular RNA was extracted using a Total RNA Extraction Kit (SM130, Sevenbio, Beijing, China), breast tissue RNA was extracted using Trizol (Invitrogen, Carlsbad, CA, USA). cDNA was produced using a one-step reverse transcription kit (MR05101, Monad, Jiangsu, China), and qPCR detection was performed using SYBR fluorescent dye (MQ00401, Monad, Jiangsu, China). Gene expression was analyzed using the 2-ΔΔCT method with β-Actin as the internal reference gene. The primers used in the experiments sequences were synthesized by Shanghai Sangong Bioengineering Co. and their sequences are shown in Tables S2 and S3.

Cellular and breast tissue proteins were extracted using RIPA buffer. The protein concentration was determined by the BCA method. The Westen blot assay was performed after mixing with loading dye. Proteins were separated by 10% SDS-PAGE and transferred to PVDF membrane. The membrane was blocked for 30 min at room temperature using protein-free rapid blocking solution; primary antibody was incubated for 2 h at room temperature; washed four times with TBST for 8 min each time; secondary antibody was incubated for 1 h at room temperature; washed four times with TBST for 8 min each time; and imaged using ECL developer solution. The primary target protein antibody was diluted at a ratio of 1:1000, and β-Actin was used as an internal reference at a dilution of 1:2000. The secondary antibody used was HRP-labeled sheep anti-rabbit IgG (1:5000).

#### Determination of triglycerides

Intracellular triglycerides content was determined using a triglyceride assay kit (E1013, Applygen, Beijing, China). The BCA method was used to determine the cellular protein concentration, and the relative quantitative analysis was performed using the ratio of triglycerides content to protein concentration.

#### BODIPY staining

Lipid droplets in BMECs were stained using BODIPY 493/503 (D2191, Thermo Fisher Scientific, Waltham, MA, USA) with reference to published literature.^44^ The general procedure was as follows: six-well plate cell crawls were prepared, and cells were processed in predetermined groupings when they were approximately 60% confluent. Subsequently, the culture medium in the well plates was discarded, and the cells were washed three times with PBS, fixed in 4% paraformaldehyde for 30 min, washed and then stained with 1 μg/mL BODIPY working solution for 25 min, washed three times with PBS and then sealed using an anti-quenching sealer containing DAPI (Beyotime, Shanghai, China). The samples were observed and analyzed using a fluorescence microscope (Olympus, Tokyo, Japan).

#### Hematoxylin-eosin (HE) staining and oil red O staining

Paraffin sections of 5 μm thickness were used for HE staining. Frozen sections of 20 μm thickness were used for Oil Red O staining. Microscopic observation of mammary gland alveoli and fat deposition was performed.

#### Immunohistochemistry

The expression FASN and KLF4 proteins was analyzed in breast tissues using an IHC assay kit (BOSTER, Wuhan, China), and the general procedure was as follows: 5 μm-thick paraffin sections were sequentially deparaffinized/rehydrated, antigenically repaired, closed, incubated with primary and secondary antibodies, photographed and observed by microscopy. The primary antibody was used at a 1:200 dilution.

#### Yeast monohybrid point-to-point validation

The FASN promoter sequence (2000 bp upstream of the transcription start site) was ligated to the pHIS2 plasmid to construct the yeast monohybrid bait vector pHIS2-FASN, and the CDS region sequence of KLF4 was ligated to the pGADT7 plasmid to construct the yeast monohybrid prey vector pGADT7-KLF4. The composition of the complete yeast medium was 1% yeast extract, 2% tryptone, 2% glucose and 0.02% adenine. Yeast Defective Screening Medium was used based on Clontech's PT3024-1/Yeast Protocols Handbook. 3AT (10 mM) was used for subsequent experiments after the self-activation assay. The yeast-deficient media used in the experiments are indicated by the following abbreviations: SD-T for tryptophan deficiency; SD-TH for tryptophan and histidine deficiency; SD-TL for tryptophan and leucine deficiency; and SD-TLH for tryptophan, histidine, and leucine deficiency. The prey vector pGAD53m and the corresponding bait vector pHIS2-P53 were cotransfected and used as positive controls, the empty vector pGADT7 and the bait vector pHIS2-FASN were cotransfected and used as negative controls, and the experimental group was that cotransfected with the prey vector pGADT7-KLF4 and the bait vector pHIS2-FASN. The single colonies that were successfully transfected in each group were resuspended in 2 mL of ddH_2_O water, the absorbance was adjusted to OD600=0.002, three concentration gradients were set, and 10 μl was collected up for the SD-TL, SD-TLH, and SD-TLH+10 mM 3AT medium plate-spotting experiments. SD-TL indicates that SD medium lacks Trp and Leu;SD-TLH indicates that SD medium lacks Trp、 Leu and His;3AT is a competitive inhibitor of yeast HIS3 protein synthesis and is used to inhibit leaky expression of the His3 gene.Each plate was spotted three times and cultured at a constant temperature of 30°C for 3-5 days. Based on the growth conditions of the colonies, we determined whether the prey and bait interact with each other.

#### Dual luciferase reporter gene assay

FASN promoter sequences were ligated into pPRO-RB-Report to construct a reporter gene vector (FASN-WT); the Jaspar database predicted the presence of multiple KLF4 protein-binding motifs in the 208-601 region upstream of the FASN transcriptional start site, and promoter sequences missing this region were ligated into pPRO-RB-Report to construct a mutant reporter gene vector (FASN-MUT). The reporter gene vector and the KLF4 overexpression vector were cotransfected into HEK293 cells, and the interaction between KLF4 and the FASN promoter region was analyzed based on the relative fluorescence value of the reporter gene.

#### Nontargeted metabolome analysis

Mouse mammary tissue was ground in liquid nitrogen and placed in Eppendorf tubes. 500 μL of 80% methanol was added to each 100 mg of tissue for homogenization, and the supernatant was centrifuged at 15000 × g for 20 min at 4°C and diluted with an appropriate amount of water until the methanol content was 53%. The supernatant was centrifuged again at 15000 × g for 20 min at 4°C, and then the supernatant was taken for LC‒MS analysis.^45^ The chromatographic parameters were as follows: HypesilGold column (C18), 40°C, flow rate of 0.2 mL/min, eluent of 0.1% formic acid and methanol in positive mode, and eluent of 5 mM ammonium acetate (pH 9.0) and methanol in negative mode. The mass spectrometry scanning range was m/z 100-1500; the ESI source settings were as follows: spray voltage: 3.2 kV; sheath gas flow rate: 40 arb; aux gas flow rate: 10 arb; and capillary temperature: 320°C. Polarity: positive; negative; MS source: 0.1% formic acid and methanol; negative mode: 5 mM ammonium acetate (pH 9.0) and methanol. Polarity: positive; negative; MS/MS secondary scans are data-dependent scans. All reagents were mass spectrometry grade. The downstream files were processed by standard procedures for subsequent bioinformatics analysis using the Kidio Cloud Platform (http://www.omicsmart.com) (accessed on 5 October 2023).

### Quantification and statistical analysis

Each experiment was repeated at least three times, and all data are expressed as the mean ± standard deviation. Western blot bands were analyzed in grayscale using ImageJ, and data were statistically analyzed and plotted using GraphPad Prism 9.5 (La Jolla, CA, USA). Comparisons between two groups were made using unpaired t-tests, and comparisons between multiple groups were made using one-way ANOVA with Tukey post hoc tests. P < 0.05 was considered statistically significant.
