# Supplementary material for: KLF4 promotes milk fat synthesis by regulating the PI3K-AKT-mTOR pathway and targeting FASN activation in bovine mammary epithelial cells
Source: iScience. 2024 Apr 30;27(6):109850. doi: 10.1016/j.isci.2024.109850 (PMC11108978; doi:10.1016/j.isci.2024.109850)
Supplement: Document S1. Tables S1–S3 [file mmc1.pdf]

iScience, Volume *27*

Supplemental information

KLF4 promotes milk fat synthesis by regulating the PI3K-AKT-mTOR pathway and targeting FASN activation in bovine mammary epithelial cells

Hong-Yu Wu, Zhong-Hao Ji, Wen-Yin Xie, Hai-Xiang Guo, Yi Zheng, Wei Gao, and Bao Yuan

Supplemental information

KLF4 promotes milk fat synthesis by regulating the PI3K-AKT-mTOR pathway and targeting FASN activation in bovine mammary epithelial cells

Hong-Yu Wu, Zhong-Hao Ji, Wen-Yin Xie, Hai-Xiang Guo, Yi Zheng, Wei Gao, Bao Yuan1

Table S1 Milk Fat Percentage and Milk Protein in High and Low Milk Fat Cows

A

	Number	milk fat (%)	milk protein (%)
Cows with low-fat milk	1	2.90	3.11
	2	3.16	3.26
	3	2.34	3.22
	4	3.03	3.52
	5	2.66	3.22
Cows with high-fat milk	6	4.31	3.52
	7	4.77	3.76
	8	3.91	3.45
	9	3.74	3.37
	10	4.15	3.86

B

Milk Components of Lactating Holstein Dairy Cows		
milk component	low-fat milk	high-fat milk
milk fat (%)	2.82±0.29	4.18±0.36
milk protein (%)	3.27±0.14	3.59±0.19

Table S2. Primers (*Bos taurus*) of RT-qPCR

gene	Primers	Length(bp)
B-KLF4	(F)TACCAAGAGCTCATGCCACC	160
	(R)GCAGGTGTGCCTTGAGATGA	
B-FASN	(F)CCTCAAGATGAAGGTGGTGCT	170
	(R)GGCCCTGGGTTATATCGAGC	
B-mTOR	(F)TTCTGACTACAGCACCAGCG	120
	(R)TGAGAGAGGGACTGGTCTCG	
B-PPARG	(F)ACTTTGGGATCAGCTCCGTG	137
	(R)GTCAGCTCTTGGGAACGGAA	
B-SCD1	(F)ACCTGGCTGGTGAATAGTGC	176
	(R)AAGTTGATGTGCCAGCGGTA	
B-SREBP1	(F)GACACCACCAGCATCAACCA	137
	(R)GCCAAGGAGAAGAGCACCAG	
B-FABP4	(F)AGCTGCACTTCTTTCTCACCT	198
	(R)GTGACCACACCCCCATTCAA	
B-ACTB	(F)CACCGCAAATGCTTCTAGGC	186
	(R)TGTCACCTTCACCGTTCCAG	

Table S3. Primers (*Mus musculus*) of RT-qPCR

Gene	Primers	Length(bp)
M-KLF4	(F)GGAGAAGACACTGCGTCCAG	178
	(R)TAGGAGGGCCGGGTTGTTAC	
M-FASN	(F)GAGGGTGTGCCATTCTGTCA	167
	(R)GCTATTCTCTACCGCTGGGG	
M-mTOR	(F)CCGCTACTGTGTCTTGGCAT	118
	(R)CAGCTCGCGGATCTCAAAGA	
M-PPARG	(F)AAGAAGCGGTGAACCACTGA	154
	(R)GGAATGCGAGTGGTCTTCCA	
M-SCD1	(F)GTACCGCTGGCACATCAACT	163
	(R)AACTCAGAAGCCCAAAGCTCA	
M-SREBF1	(F)CTGGTGAGTGGAGGGACCAT	161
	(R)GACCGGTAGCGCTTCTCAAT	
M-FABP4	(F)GGATTTGGTCACCATCCGGT	161
	(R)TTCCATCCCACTTCTGCACC	
M-ACTB	(F)TGAGCTGCGTTTTACACCCT	198
	(R)GCCTTCACCGTTCCAGTTTT	
